# Antibacterial Activity of Traditional Medicinal Plants: Combating Antibiotics Resistance in Animal Wound Infections

**DOI:** 10.1002/vms3.70361

**Published:** 2025-05-06

**Authors:** Dan Jia, Safia Arbab, Hanif Ullah, Khalid J. Alzahrani, Fuad M. Alzahrani, Khalaf F. Alsharif, Jiyu Zhang, Ka Li

**Affiliations:** ^1^ Outpatient Department West China Hospital, Sichuan University/West China School of Nursing, Sichuan University Chengdu China; ^2^ Lanzhou Institute of Husbandry and Pharmaceutical Sciences Chinese Academy of Agricultural Sciences Lanzhou China; ^3^ Medicine and Engineering Interdisciplinary Research Laboratory of Nursing and Materials/Nursing Key Laboratory of Sichuan Province West China Hospital, Sichuan University/West China School of Nursing Sichuan University Chengdu Sichuan China; ^4^ Department of Clinical Laboratories Sciences College of Applied Medical Sciences Taif University Taif Saudi Arabia

**Keywords:** animal wound, antibiotics, antimicrobial, herbal medicinal plants

## Abstract

The rise of antibiotic resistance poses a significant challenge to veterinary medicine, particularly in the treatment of wound infections in animals. This study aimed to evaluate the antibacterial properties of traditional medicinal plants against four bacterial strains isolated from septic animal wound infections and to compare their effectiveness with broad‐spectrum antibiotics. A total of 150 bacterial isolates were collected using sterile cotton swabs, cultured on nutrient and mannitol salt agar for growth and identified through catalase and coagulase tests. The study assessed antibiotic resistance and tested the bacterial isolates’ susceptibility to medicinal plants using the disc diffusion method. Among the isolates, *Staphylococcus aureus* was the most common pathogen, accounting for 26.6% (40 isolates), followed by *Escherichia coli* at 26% (39 isolates). *Streptococcus* spp. and *Pseudomonas* spp. were found in 20% (30 isolates) and 16% (25 isolates), respectively. Antibiogram analysis revealed notable antibiotic resistance, with *S. aureus* showing the highest resistance to Ciprofloxacin (60.5%) and Rifampicin (57.5%). *E. coli* exhibited 61.5% resistance to Ciprofloxacin and 56.4% to streptomycin. *Streptococcus* spp. had the highest resistance to Ciprofloxacin (53.3%), whereas *Pseudomonas* spp. showed the greatest resistance to Chloramphenicol (52%). Ethanol extracts of the medicinal plants, prepared using various solvents, were utilized for testing. Ethanol extracts of *Loranthus acaciae* and *Cymbopogon proximus* at concentrations of 60–90 µL demonstrated the largest inhibition zones, ranging from 55.5 ± 3.85 to 57.5 ± 2.5 mm against *E. coli* and *S. aureus*. Inhibition zones for *Streptococcus* spp. and *Pseudomonas* spp. ranged from 50 ± 2 to 48.3 ± 1.7 mm. In comparison, the standard antibiotics exhibited inhibition zones of 58.95 ± 2.55–60 ± 2.5 mm against *S. aureus* and *E. coli*, with slightly lower zones (51.65 ± 1.6–49 ± 1 mm) observed for *Streptococcus* spp. and *Pseudomonas* spp. These findings underscore the significant antibacterial activity of medicinal plants against multidrug‐resistant pathogens in animal wound infections, highlighting their potential to accelerate healing, reduce infection severity and provide a cost‐effective alternative to combat antibiotic resistance.

## Graphical abstract

This study addressed the growing issue of antibiotic resistance in veterinary wound infections by evaluating the antibacterial activity of traditional medicinal plants. A total of 150 bacterial isolates were collected, with *Staphylococcus aureus* (26.6%) and *Escherichia coli* (26%) being the most prevalent. High resistance to ciprofloxacin and other antibiotics was observed among the isolates. Ethanol extracts of *Loranthus acacia* and *Cymbopogon proximus* showed strong antibacterial effects, with inhibition zones comparable to standard antibiotics. The results highlight the potential of medicinal plants as effective, low‐cost alternatives to treat multidrug‐resistant infections in animals.

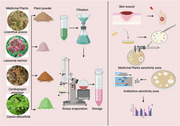

**Abbreviations**
AMX: Amoxicillin
APX: Ampicillin
AU: Augmentin
CEP: Cephalexin
CH: Chloramphenicol
CN: Gentamicin
CPX: Ciprofloxacin
E: Erythromycin
LEV: Levofloxacin
NA: nalidixic acid
NB: Norfloxacin
OFX: Ofloxacin
PEF: Pefloxacin
PN: Ampicillin
RD: Rifampicin
Rest: resistant
S: streptomycin
SXT: sulphamethoxazole‐trimethoprim

## 1 Introduction

Addressing wounds and their associated challenges has become a significant global healthcare concern, imposing substantial financial burdens on healthcare systems. Wound healing is a complex and carefully orchestrated biological process that begins immediately following tissue injury (Ullah et al. 2024). Wound infections in animals often harbour resistant bacterial strains, reducing the efficacy of conventional antibiotics and necessitating alternative therapeutic approaches (Van Boeckel et al. 2019). Traditional medicinal plants have been used for centuries to treat infections, and recent studies have highlighted their potential antibacterial properties (Arbab et al. 2021d).

Antibiotic resistance has become a global health concern, affecting both human and veterinary medicine. The overuse and misuse of antibiotics in treating bacterial infections have led to the emergence of multidrug‐resistant pathogens, complicating the management of infectious diseases in animals (Catalano et al. 2022). Wound infections in animals often harbour resistant bacterial strains, reducing the efficacy of conventional antibiotics and necessitating alternative therapeutic approaches (Mba and Nweze 2022).

Antibiotics have played a crucial role in controlling bacterial infections; however, their overuse and misuse in animals have contributed to the rise of resistant bacterial strains. Common wound pathogens, such as *Staphylococcus aureus*, *Pseudomonas aeruginosa* and *Escherichia coli*, have developed resistance mechanisms that compromise treatment effectiveness (Laxminarayan et al. 2016). The challenge of antibiotic resistance in animal health is particularly alarming because resistant bacteria can spread to humans through direct contact, contaminated food or the environment, posing a serious zoonotic threat (Arbab et al. 2025; Zhang et al. 2024). Given these concerns, alternative treatments such as medicinal plant extracts have gained attention for their ability to combat bacterial infections without contributing to resistance.

Medicinal plants contain bioactive compounds such as flavonoids, tannins, alkaloids and saponins, which have demonstrated antibacterial effects against a range of pathogens (Gurib‐Fakim 2006). Their ability to inhibit bacterial growth and disrupt biofilm formation makes them promising candidates for alternative antimicrobial therapies (Sahoo et al. 2021). Several studies have shown that plant extracts exhibit significant antibacterial activity against both Gram‐positive and Gram‐negative bacteria, including common wound pathogens such as *S. aureus*, *P. aeruginosa* and *E. coli* (Liu et al. 2023; Matsuura and Barg 2013). These organisms can survive for long periods of time because they are naturally resistant to a variety of antibiotics and antiseptics. They are able to colonize injured skin and multiply with little nourishment (Gao et al. 2023; Olayinka et al. 2004). Infectious diseases contribute to high morbidity and mortality rates globally. Every year, millions of people die due to the rise of antibiotic‐resistant bacterial strains, particularly in intensive care units (ICUs) (Angel 2016).

Medicinal plants contain bioactive compounds, such as flavonoids, tannins, alkaloids and saponins, which have demonstrated antibacterial effects against a range of pathogens (Gurib‐Fakim 2006). Their ability to inhibit bacterial growth and disrupt biofilm formation makes them promising candidates for alternative antimicrobial therapies (Arbab et al. 2024; Ohalete et al. 2012; Özaydın and Aydın 2023).

Although conventional antibiotics remain the primary choice for treating bacterial infections, their decreasing effectiveness due to resistance calls for comparative studies with alternative agents. Some medicinal plant extracts have been found to exhibit antibacterial activity comparable to that of broad‐spectrum antibiotics (Dhama et al. 2014). Evaluating the efficacy of medicinal plant extracts against bacterial strains isolated from septic animal wound infections is crucial in determining their potential as viable alternatives. By comparing the antibacterial activity of plant extracts with commonly used antibiotics, this study aims to assess their effectiveness in controlling wound infections in veterinary medicine.

This study aims to evaluate the antibacterial activity of selected traditional medicinal plants against bacterial strains isolated from septic animal wound infections. Additionally, their effectiveness will be compared with broad‐spectrum antibiotics to explore their potential as alternative treatments in veterinary medicine.

## 2 Materials and Methods

### 2.1 Ethic Statement

All animal experiments were carried out in accordance with the regulations regarding the use of animals in toxicology. The research focused on Antibacterial Activity of Traditional Medicinal Plants: Combating Antibiotics Resistance in Animal Wound Infections. The Animal Administration and Ethics Committee of the Chinese Academy of Agricultural Sciences' Lanzhou Institute of Husbandry and Pharmaceutical Sciences gave approval to all the experiments. CARS‐37 was the certificate number.

### 2.2 Sample Collection

A total of 150 wound isolates were collected from donkeys, cattle and buffalo on an animal farm. Wound exudates were obtained using sterile cotton swabs from infected areas of each animal and transferred to freshly prepared nutrient agar and mannitol salt agar (Oxoid) slants. Following standard bacteriological procedures, the samples were cultured on various media, including mannitol salt agar, 5% sheep blood agar and chocolate agar. The cultures were then incubated at 37°C for 24–48 h (Chukwuma et al. 2015).

### 2.3 Identification of Bacterial Isolates, Culture Media and Species

The bacterial isolates were identified using standard microbiological techniques. Samples collected from infected wounds were cultured on selective and differential media to facilitate bacterial growth and identification. Mannitol salt agar was used for the isolation of *Staphylococcus* species, whereas 5% sheep blood agar and chocolate agar supported the growth of a wide range of bacterial pathogens, including *Streptococcus* and *Pseudomonas* spp., *E. coli* and *S. aureus*.

After incubation at 37°C for 24–48 h, bacterial colonies were examined for morphological characteristics such as shape, colour and haemolytic patterns. Gram staining was performed to differentiate Gram‐positive from Gram‐negative bacteria. Further biochemical tests, including catalase, coagulase, oxidase and motility tests, were conducted to confirm bacterial species.

Commonly identified bacterial species included *S. aureus*, *P. aeruginosa* and *E. coli*. These pathogens are frequently associated with wound infections in animals and are known for their resistance to multiple antibiotics (Arbab et al. 2021d).

### 2.4 The Bacterial Inoculum's Preparation

Pure bacterial isolates were introduced into nutrient broth and incubated at 37°C for up to 5 h, or until the turbidity reached the 0.5 McFarland standard on the turbidity scale. The McFarland standard was calibrated by mixing 9.6 mL of a 1% barium chloride solution with 0.4 mL of 1% sulphuric acid, resulting in an approximate bacterial density of 1.2 × 10^9 CFU/mL (Cheesbrough 2005).

### 2.5 Antimicrobial Susceptibility Testing Profile Determination

Following the criteria established by the Clinical and Laboratory Standards Institute (CLSI), antimicrobial susceptibility testing was carried out on Mueller‐Hinton agar (MHA) using the Kirby‐Bauer disc diffusion method. The results were interpreted in accordance with CLSI standards (Wayne 2011). Animal wound infection isolates were tested for antibiotic resistance using the disc diffusion method. Overnight bacterial cultures were diluted in sterile 0.9% sodium chloride solution and adjusted to the 0.5 McFarland standard. The bacterial suspensions were then evenly spread onto MHA to create a uniform lawn. Commercially available antibiotic discs were placed on the agar surface, ensuring adequate spacing between them. The plates were then incubated at 37°C for 18–24 h. After incubation, the zones of inhibition were measured using a ruler or calliper to assess the bacterial susceptibility to the tested antibiotics (Kos et al. 2006). In accordance with reference standards, sensitivity, intermediate sensitivity and resistance were evaluated using the zone of total growth inhibition surrounding each disc. Amoxicillin, Gentamicin, Levofloxacin, Ciprofloxacin, Streptomycin, Rifampicin, Norfloxacin, Sulfamethoxazole‐trimethoprim, Ampicillin, Cephalexin, Nalidixic acid and Ofloxacin were among the antibiotic discs used in the test.

### 2.6 Crude Medicinal Plant Extract

Crude medicinal plant extracts are derived directly from plant materials and are often used in traditional medicine for their therapeutic properties. Plant material was collected from a nursery, dried and finely ground into a powder using a mortar and pestle. One kilogram of the powdered plant material was then soaked in 3 L of 90% ethanol. The extraction process was carried out over 3 days using a rotary evaporator, with daily filtration and the evaporation of the ethanol solvent under reduced pressure (Moglad et al. 2020). The ‘crude medicinal plant extract’ refers to unrefined extracts containing a mixture of bioactive compounds obtained through solvent extraction (methanol or aqueous methods). This approach was used to retain the synergistic effects of multiple active components, which can enhance the antibacterial and wound‐healing efficacy compared to isolated compounds (Berida et al. 2024).

### 2.7 Fractionation of Extract From Ethanol

The process begins with the preparation of a crude extract by macerating or refluxing plant material in ethanol, which effectively solubilizes a wide range of phytochemicals, including phenolics, flavonoids, alkaloids and terpenoids (Hussain et al. 2024).

Ethanol extract was fractionated by liquid–liquid fractionation method (Handa et al. 2008). In brief, 20 g of the extract was dissolved in 500 mL of distilled water and partitioned three times with an equal volume of *n*‐hexane. The aqueous layer was then lyophilized using a freeze dryer. The *n*‐hexane layer was next partitioned with 90% ethanol (MeOH) in water (H_2_O), and the *n*‐hexane layer was separated. The MeOH layer's concentration was then reduced from 90% to 80% by adding distilled water. Finally, fractionation of the MeOH layer was performed using dichloromethane as the solvent.

### 2.8 Antimicrobial Activities of Medicinal Plant Extracts

A bacterial suspension was prepared from fresh subcultures of all tested strains, which had been grown overnight on nutrient agar. The direct colony suspension method was used, where two to three colonies were transferred into sterile 0.9% normal saline solution. The turbidity of the suspension was adjusted to match the 0.5 McFarland standard, ensuring a bacterial cell concentration of approximately 1–2 × 10^8 CFU/mL for each tested strain.

### 2.9 Antibacterial Susceptibility: Well‐Diffusion Technique

The antibacterial activities of the ethanol extract and fractions of the medicinal plant were evaluated using the agar well‐diffusion method (Balouiri et al. 2016). Taking into consideration the recommendations by Eloff (2019), to ensure accurate screening of antimicrobial activity, 200 mL of each bacterial isolate was evenly spread on MHA plates using sterile cotton swabs. Wells, 8 mm in diameter, were then made in the agar using a sterile cork borer. A stock solution of the plant extract and fractions, prepared at 100 mg/mL in 100% dimethyl sulphoxide (DMSO), was used for antibacterial testing. The extract was subsequently diluted with methanol to achieve various concentrations. Volumes of 60 and 90 µL of each extract and fraction were added to three separate wells on each plate, with methanol serving as a negative control. The plates were left uncovered in a sterile safety cabinet for 20 min to allow the solutions to dry (Dkhil et al. 2020). The results were recorded by measuring the size of the inhibition zone after 24 h of incubation at 35°C ± 2°C. The experiments were conducted in duplicate and repeated three times independently. The results are presented as the mean inhibition zone ± standard deviation (Ali et al. 2018; Mujeeb et al. 2014).

### 2.10 Data Analysis

Microsoft Office Excel 2007 was used to perform descriptive statistics and provide a graphical representation of the data. Descriptive statistics (mean ± SD), frequency and percentage analysis were used to present the study's data. These methods are appropriate as they allow for effective comparison of multiple groups and determination of significant differences in antimicrobial efficacy.

## 3 Results

### 3.1 Confirmation of Bacterial Pathogens in Animal Wound Infection

The overall prevalence of bacterial pathogens in animal wound infections was determined by analysing 150 wound samples, all of which tested positive for various microorganisms. The prevalence rates of these pathogens are shown in Table 1. Among the 150 wound swab samples, *S. aureus* was the most frequently identified bacterial species, found in 40 samples (26.6%), followed by *E. coli* in 39 samples (26%), *Streptococcus* spp. in 30 samples (20%) and *Pseudomonas* spp. in 25 samples (16.6%). The identification of these organisms was based on their morphological and cultural characteristics, staining reactions and further confirmed through biochemical tests. The most common infections identified were caused by *S. aureus* and *E. coli*, found in 40 (26.6%) and 39 (26%) of the infected wounds, respectively.

**TABLE 1:** Prevalence of bacterial isolated from wound infection of animals.

Bacterial species	Total number of organisms	Total percentage
*Staphylococcus aureus*	40	26.6
*Escherichia coli*	39	26
*Streptococcus* spp.	30	20
*Pseudomonas* spp.	25	16.6

### 3.2 Broad‐Spectrum Antibiotics’ Antibacterial Activity Against Isolated Organisms

On the basis of the data provided, the antibiotic resistance of various bacterial species can be assessed by the zone of inhibition. *S. aureus* shows high resistance to Ciprofloxacin and Rifampicin, with zone sizes of 62.5 and 57.5 mm respectively. *S*. spp. exhibits the same high resistance to AMX and streptomycin with a zone size of 61.5 and 56.4 mm. *E. coli* shows higher resistance to Ciprofloxacin and Rifampicin with zones of inhibition of (61.5 and 56.4 mm), respectively, which are lower compared to other antibiotics *Pseudomonas* spp. (52 and 48 mm), Ciprofloxacin and Rifampicin, respectively.

**FIGURE 1:**
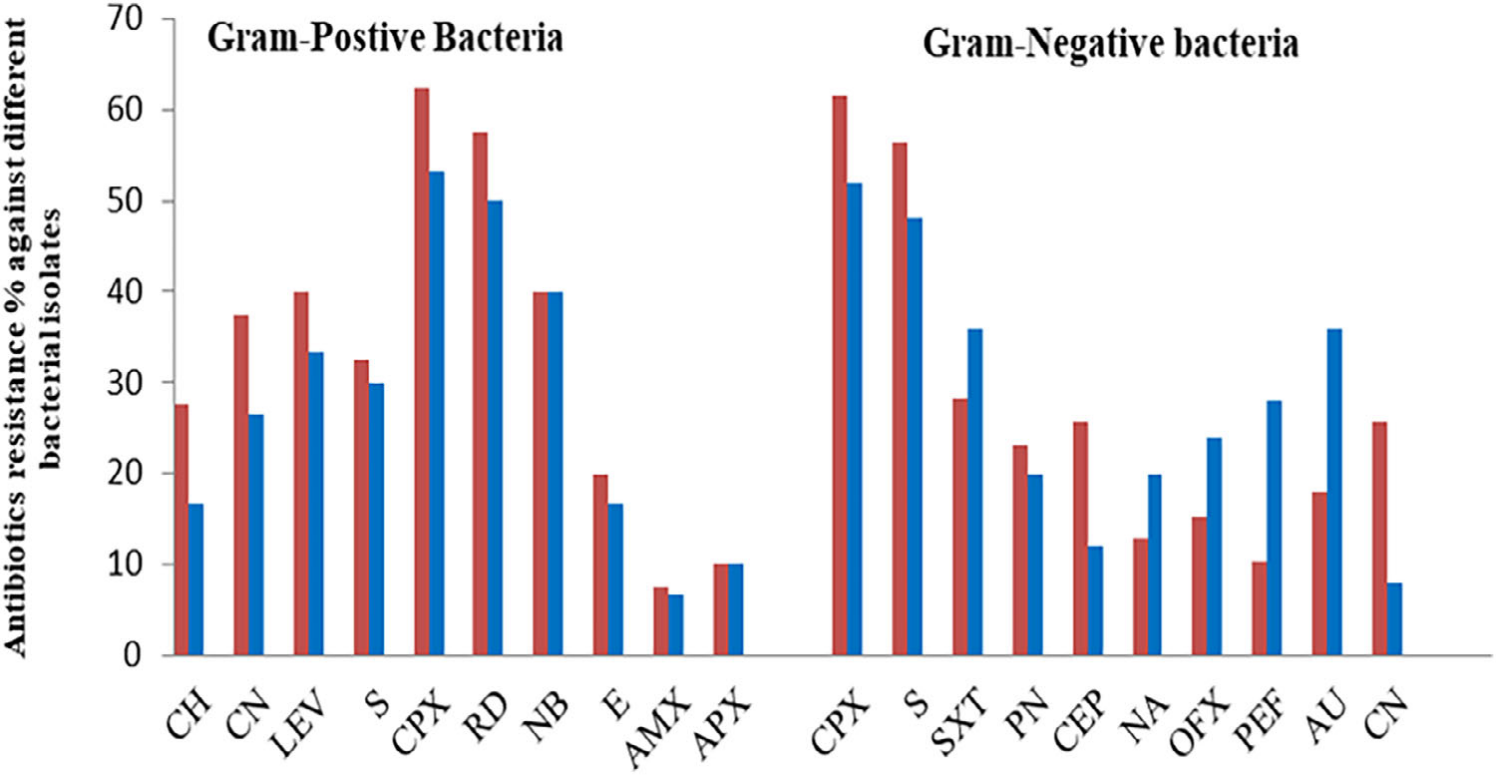
The most prevalent bacterial isolates from the wounds of diseased animals exhibit varying percentages of antibiotic resistance.

These findings highlight significant contamination levels with varying resistance rates among the bacterial isolates. The most identified organism overall was Ciprofloxacin and Rifampicin, which show the highest resistance in *S. aureus* and *E. coli*. exhibited a notably high resistance rate to broad‐spectrum antibiotics, as shown in Table 2 and Figure 1.

**TABLE 2:** The prevalent Gram‐positive and Gram‐negative bacterial isolates in animal wound infections and their antibiotic resistance patterns assessed.

Bacterial species	CH	CN	LEV	S	CPX	RD	NB	E	AMX	APX	SXT	PN	CEP	NA	OFX	PEF	AU
*Staphylococcus aureus* (*n* = 40)	27.5	37.5	40.0	32.5	62.5	57.5	40.0	20.0	7.5	10.0	—	—	—	—	—	—	—
*Streptococcus* spp. (*n* = 30)	16.6	26.6	33.3	30.0	53.3	50.0	40.0	16.6	6.6	10.0	—	—	—	—	—	—	—
*Escherichia coli* (*n* = 39)	—	25.6	—	56.4	61.5	—	—	—	—	—	28.2	23.0	25.6	12.8	15.3	10.2	17.9
*Pseudomonas* spp. (*n* = 25)	—	8.0	—	48.0	52.0	—	—	—	—	—	36.0	20.0	12.0	20.0	24.0	28.0	36.0

Abbreviations: AMX, Amoxicillin; APX, Ampicillin; AU, Augmentin; CEP, Cephalexin; CH, Chloramphenicol; CN, Gentamicin; CPX, Ciprofloxacin; E, Erythromycin; LEV, Levofloxacin; NA, nalidixic acid; NB, Novobiocin; OFX, Ofloxacin; PEF, Pefloxacin; PN, Penicillin; RD, Rifampin; S, streptomycin; SXT, Trimethoprim‐sulfamethoxazole.

### 3.3 Antibacterial Activity of Medicinal Plant Extracts and Fractions

**FIGURE 2:**
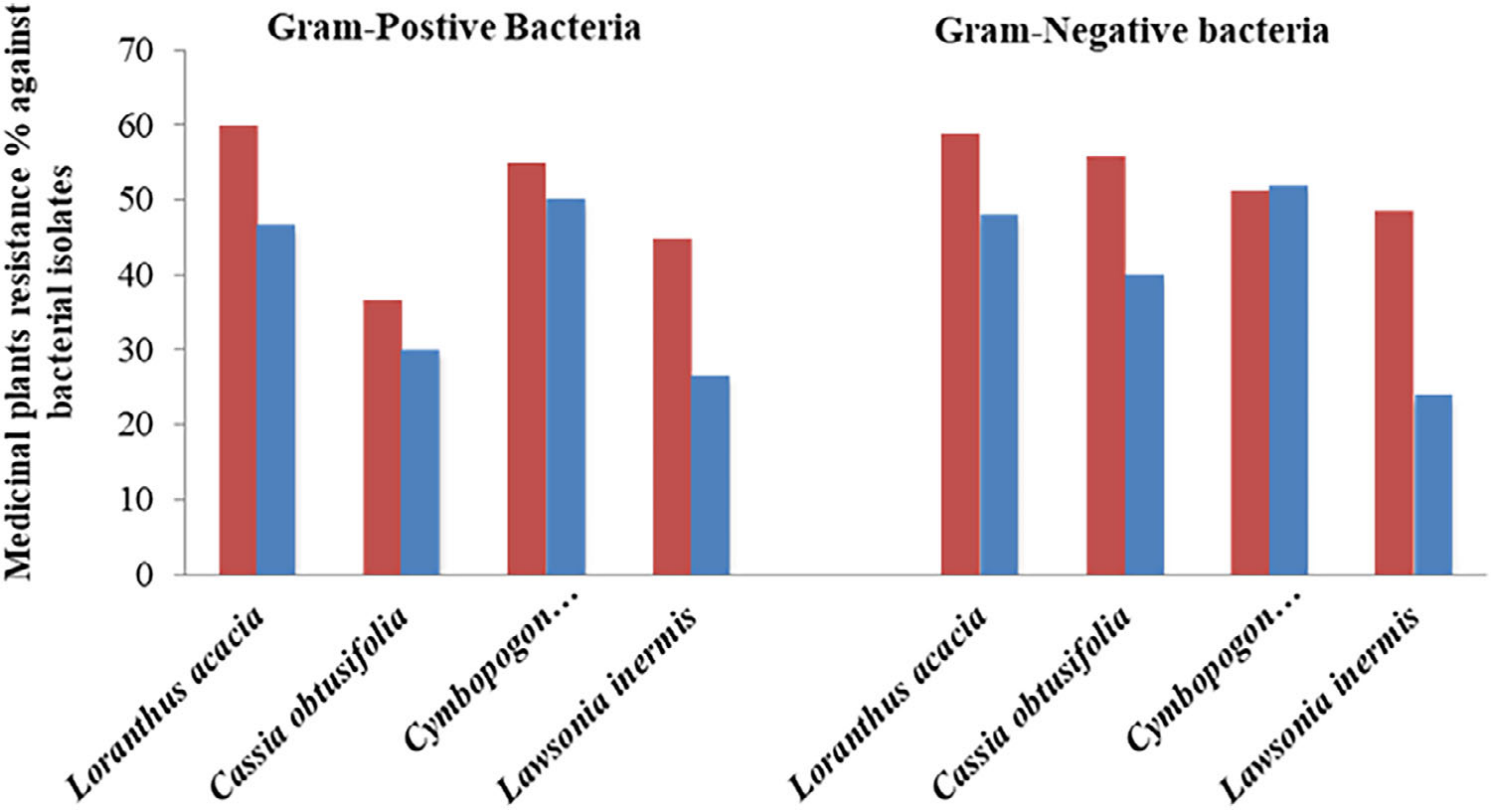
Bacteria from animal wound infections show different resistance levels to medicinal plants.

This study investigated the antimicrobial properties of medicinal plant extracts (60–90 µL) against several common pathogenic bacteria. Using the well‐diffusion method, significant zones of inhibition were observed across all tested pathogens. The antibacterial potency was assessed by measuring the inhibition zone diameters. Ethanol extracts of *Loranthus acaciae* and *Cymbopogon proximus* demonstrated the strongest antibacterial activity, with maximum efficacy recorded at 90 µL. These extracts were particularly effective against both Gram‐positive and Gram‐negative bacteria, including *S. aureus* (46.6%, 50%), *Streptococcus* spp. (60%, 55%), *E. coli* (58.9%, 51.2%) and *Pseudomonas* spp. (48%, 52%). Although some bacterial strains exhibited resistance near the discs, the ethanol extracts showed a higher susceptibility against Gram‐negative isolates. A detailed summary of the findings is provided in Table 3 and Figure 2.

**TABLE 3:** Zone of inhibition and resistance patterns of medicinal plant extracts against common bacterial organisms from animal wound infections.

	Susceptibility zones (mm) of different medicinal plant extracts against microbial species							
	*Loranthus acaciae*		*Cassia obtusifolia*		*Cymbopogon proximus*		*Lawsonia inermis*	
Microbial spp. Isolates No.:	90 µL Rest	60 µL Rest	90 µL Rest	60 µL Rest	90 µL Rest	60 µL Rest	90 µL Rest	60 µL Rest
*Escherichia coli* (*n* = 39)	58.9	28.2	35.8	33.3	51.2	48.7	48.6	23.0
*Staphylococcus aureus* (*n* = 40)	60	35.7	36.6	34.8	55	53.2	44.9	35.7
*Streptococcus* spp. (*n* = 30)	46.6	33	30	26.6	50	50	26.6	20
*Pseudomonas* spp. (*n* = 25)	48	36	40	24	52	48	24	20

The results of this study clearly indicate that *S. aureus* and *E. coli* exhibited the highest resistance to the medicinal plant extracts, particularly with the 90 µL ethanol extract of *L. acaciae* and *C. proximus*.

### 3.4 Evaluation of Medicinal Plant Ethanol Extracts’ Antibacterial Activity Against Common Pathogenic Bacteria in Comparison to Standard Antibiotics

**FIGURE 3:**
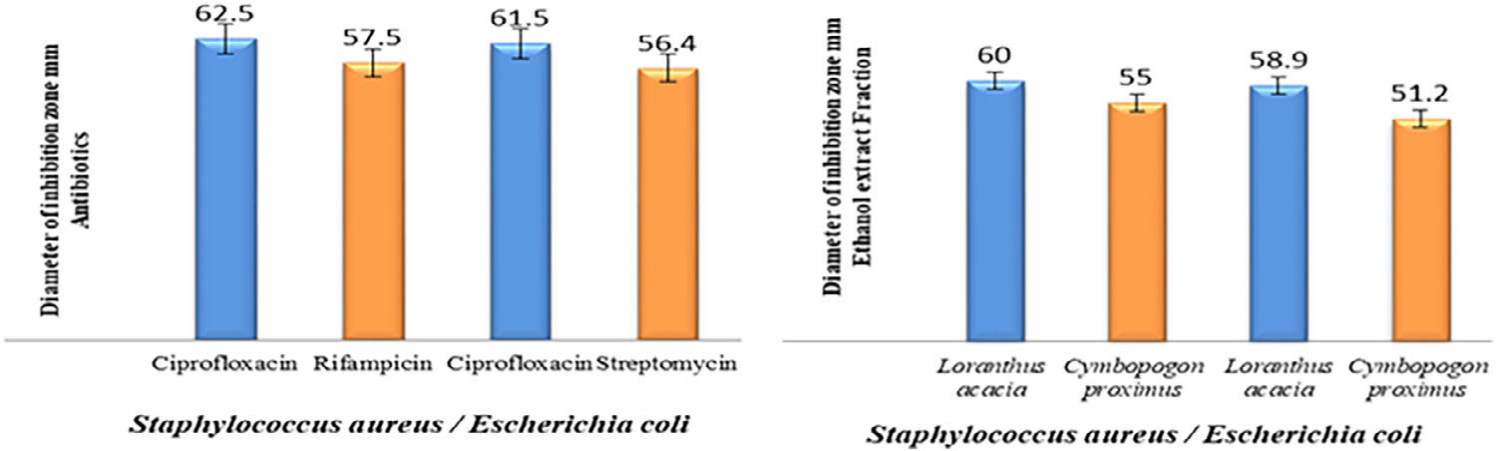
This figure illustrates the antimicrobial activity of ethanol extracts at 60 and 90 µL concentrations against common bacterial strains and standard antimicrobial drugs. Both *Staphylococcus aureus* and *Escherichia coli*, known for broad‐spectrum resistance, showed considerable resistance to the extracts. Experiments were conducted in duplicate and repeated three times, with results expressed as the mean inhibition zone ± standard deviation.

The antibacterial activity of medicinal plant crude and ethanol extracts was evaluated against common pathogenic bacteria and compared to standard antibiotics. The results revealed varying resistance patterns, with some bacterial species showing significant susceptibility to plant extracts. This highlights the potential of medicinal plants as alternative antimicrobial agents in combating resistant bacterial infections as shown in Table 4 and Figure 3.

**TABLE 4:** Antibiotic resistance patterns of bacterial species treated with ethanol extracts of medicinal plants.

Bacterial species	*Loranthus acaciae* (90 µL) (%)	*Cymbopogon proximus* (90 µL) (%)	Most resistant antibiotic
*Escherichia coli*	58.9	51.2	CPX (61.5%)
*Staphylococcus* spp.	60	55	CPX (62.5%)
*Pseudomonas* spp.	48	52	CPX (50%)
*Streptococcus* spp.	46.6	50	CPX (53.3%)

It is important to note that the susceptibility tests for all bacterial strains showed only a slight difference between the broad‐spectrum antibiotics and the ethanol extract fractions of the medicinal plants. This suggests that multiple active components in the plant extracts may contribute to their synergistic, multi‐target antibacterial effects.

### 3.5 Standard Resistance Rates With Medicinal Plants and Antibiotics Have Varied Means and Standard Divisions

A comparison of resistance zone diameters for different antibiotics and the four medicinal plant extracts against common Gram‐positive and Gram‐negative bacteria revealed that both standard antibiotics and the extracts of *L. acaciae* and *C. proximus* exhibited high resistance to *E. coli*, *S. aureus*, *Streptococcus* spp. and *Pseudomonas* spp. The mean ± SD zone sizes for both the antibiotics and medicinal plant extracts demonstrated greater resistance in *E. coli* and *S. aureus*, which are known to be multi‐resistant, followed by *Streptococcus* spp. and *Pseudomonas* spp., where the zone sizes indicated relatively lower resistance. There was minimal difference in the mean ± SD zone sizes for *S. aureus* and *E. coli*, as shown in Table 5.

**TABLE 5:** The bacterial organisms isolated from antibiotics and medicinal plants have varied means and standard divisions.

Bacterial spp.	Medicinal plants	Mean ± SD	Most resistance antibiotic	Mean ± SD
*Escherichia coli*	*Loranthus. acaciae* + *Cymbopogon. proximus*	55.5 ± 3.85	CPX, S	58.95 ± 2.55
*Staphylococcus* spp.	*L. acaciae* + *C. proximus*	57.5 ± 2.5	CPX, RD	60 ± 2.5
*Pseudomonas* spp.	*L. acaciae* + *C. proximus*	50 ± 2	S, CPX	49 ± 1
*Streptococcus* spp.	*L. acaciae* + *C. proximus*	48.3 ± 1.7	CPX, RD	51.6 ± 1.65

## 4 Discussion

Wounds are a leading cause of death globally, often resulting from infections due to the complex interaction between the host and pathogen. When the pathogen load surpasses the host's immune capacity, it triggers a systemic immune response. This study examines the effects of broad‐spectrum antibiotics and plant‐based medications, or phytomedicine, known for their wide range of biological activities. Herbal medicine, rich in secondary metabolites, has been traditionally used to treat both acute and chronic conditions. The study highlights the role of herbal medicines as antibacterial agents in wound care, particularly in managing animal wound infections, and underscores the importance of secondary metabolites in combating bacterial infections in animals.

Four bacterial species were identified from infected wound samples, *S. aureus*, *E. coli*, *Streptococcus* spp. and *Pseudomonas* spp., which were the most common pathogens found in animal wound infections. The incidence rates for these bacteria were 29.8%, 29.1%, 18.6% and 22.3%, respectively. Similar studies conducted globally have reported high rates of bacterial isolation from animal wound samples, and the findings of this study align with these previous reports (Arbab et al. 2020, 2021a, 2022a, 2021b, 2022b, 2021c, 2021d). The prevalence of bacterial combinations and interactions has been crucial, even outside the presence of pathogens. Our findings thus support the highest prevalence that is often found in both acute and chronic wounds (Percival et al. 2010).

Antibiotic resistance has remained a major issue in veterinary medicine, livestock management and animal husbandry (Arbab et al. 2022a; Hassan et al. 2024). Because animal germs are frequently the cause of wound infections, it is imperative to assess their resistance profiles. To evaluate the medication resistance profiles of the isolates from these animals, we obtained samples of wound infections from a variety of animals (Arbab et al. 2022b; Lawson 2008). The disc diffusion method was employed in this study to test for antibiotic sensitivity. One important discovery was that animals had several drug‐resistant strains of *S. aureus* and *E. coli* that were resistant to standard antibiotics such as Ciprofloxacin (CPX), Streptomycin (S), Rifampicin (RD) and Norfloxacin (NB). This finding supports earlier research showing a concerning rise in antibiotic resistance, especially in *E. coli* strains recovered from cattle and other animals (Wang et al. 2022; Javed et al. 2024; Lawson 2008). In another research study, it was confirmed that *S. aureus* and *E. coli* were the predominant species infecting animal wounds (Mohammed et al. 2013). This result is consistent with prior research that found a high rate of *E. coli* resistance in Jordanian cows (Motayo et al. 2013). These results are in line with recent studies that found *E. coli* to be the predominant species among animals in good health (Oloso et al. 2018).

The growing prevalence of antibiotic‐resistant bacteria in veterinary medicine has created an urgent need for alternative treatment strategies, particularly for managing wound infections in animals. Traditional medicinal plants, known for their diverse bioactive compounds, present a promising solution to this challenge. These plants produce secondary metabolites such as alkaloids, flavonoids, tannins and essential oils, which exhibit potent antibacterial properties. Studies have demonstrated the efficacy of plant extracts inhibiting the growth of common pathogens, including multidrug‐resistant strains of *S. aureus* and *P. aeruginosa* (Negi and Mirza 2020).

Medicinal plants have been studied for various biological activities, such as antioxidant, anti‐inflammatory, wound‐healing and antibacterial properties (Arbab et al. 2022b; Lawson 2008). However, this is the first study to report its antibacterial activity against clinically isolated bacteria. Additionally, our results are in line with several other studies that have highlighted the antimicrobial properties of medicinal plants against a broad range of bacteria.

Natural products offer a variety of lead compounds that could facilitate the development of new antimicrobial agents, especially as traditional antimicrobial drugs lose their effectiveness due to the rise of resistance. The secondary metabolites found in medicinal plants may employ various antimicrobial mechanisms, helping to counteract the development of resistance (Baz et al. 2024; Tadesse et al. 2016). It is worth noting that the susceptibility tests of all bacterial strains revealed only minor differences between the medicinal plant ethanol extract fractions (60–90 µL) of *L. acaciae* and *C. proximus*, which demonstrated the highest inhibition against *S. aureus* (60%–55%) and *E. coli* (51.2%–58.9%). *E. coli* and *S. aureus*, both known for their multi‐drug resistance, were highly susceptible to these extracts. This suggests that the extracts may contain multiple active compounds that could work synergistically, targeting various mechanisms of bacterial action (Bouari et al. 2011; Javed et al. 2024). A previous study indicated that the presence of multiple compounds may enhance the activity within the host, as demonstrated by the ethanol extracts of medicinal plants, which showed the highest inhibition against *S. aureus* (Manivannan et al. 2015; Paiano et al. 2020; Semwal et al. 2014).

The antibacterial qualities of *L. acacia* and *C. proximus* may be attributed to the presence of alkaloids, flavonoids, tannins, phytosterols, triterpenoids and other compounds in the extracts found by phytochemical screening of medicinal plants. Loranthus, flavanocoumarin, quercetin, catechin, rutin, methyl gallate and gallic acid were among the chemicals in plants that showed significant antibacterial action, according to another study (Badr et al. 2013). Probably because of the phytochemical components present, the plant extracts and their fractions showed efficacy against every isolate. These substances may act as β‐lactamase inhibitors, bind to active sites, affect outer membrane permeability and prevent multidrug resistance pumps for efflux (Krol et al. 2015; Mingeot‐Leclercq and Dacout 2016).

When comparing the zone diameters of broad‐spectrum antibiotics and herbal medicines in this study, it was found that there was a minimal statistically significant difference. The results showed that *L. acacia* and *C. proximus* had resistance levels of ±55.5 to 3.85 SD against *S. aureus* and ±57.5 to 2.5 SD against *E. coli*. In comparison, broad‐spectrum antibiotics such as CPX, S and RD showed resistance of ±60 to 2.5 SD against *S. aureus* and ±58.95 to 2.55 SD against *E. coli*. These findings indicated only a slight difference in resistance between *S. aureus* and *E. coli*. Similar studies globally have reported high antibacterial activity against *S. aureus* (with a zone of inhibition of 8.0 mm) but comparatively less activity against *E. coli* (Arbab et al. 2022b; Arbab et al. 2021d; Zhang et al. 2018). The findings of this study indicate that broad‐spectrum antibiotics are effective against *S. aureus* and *E. coli*, which are high‐priority pathogens responsible for wound infections in animals. Additionally, the ethanol extract demonstrated 100% efficacy and susceptibility against both Gram‐positive and Gram‐negative bacterial isolates (Matu and Van Staden 2003). A similar study was conducted, and the ethanol extract exhibited the most significant effect against *S. aureus* and *E. coli* (Rudrangshu et al. 2015). The acute toxicity of ethanol extracts from medicinal plants used to treat animal wound infections was examined in our newly published study. The findings showed that the extracts have antibacterial qualities like those of broad‐spectrum antibiotics and are non‐toxic. These results pave the way for additional in vitro and in vivo veterinary research.

This study aims to evaluate the antibacterial activity of extracts from selected medicinal plants against common wound‐infecting bacteria in animals. By assessing their efficacy, this research contributes to the growing body of evidence supporting plant‐based antimicrobials as potential alternatives or complementary treatments in veterinary medicine.

## 5 Conclusion

The increasing prevalence of antibiotic‐resistant bacteria in animal wound infections highlights the urgent need for alternative antimicrobial strategies. Traditional medicinal plants, rich in bioactive compounds, offer promising antibacterial potential against resistant pathogens such as *S. aureus* and *E. coli*. This study demonstrated that ethanol extracts from selected medicinal plants exhibit measurable antibacterial activity, reinforcing their potential as natural alternatives or complementary agents to conventional antibiotics. Although these findings support the use of plant‐based antimicrobials in veterinary medicine, further research is needed to identify the specific active compounds, optimize extraction methods and assess their safety and efficacy in vivo. Integrating traditional medicinal plants into veterinary healthcare could help mitigate antibiotic resistance while promoting sustainable and effective infection control in animal wound management.

## Author Contributions

**Safia Arbab**: manuscript writing, conceptualization, formal analysis. **Dan Jia**: funding acquisition, review and editing. **Hanif Ullah, Khalid J. Alzahrani, Fuad M. Alzahrani, Khalaf F. Alsharif**: writing‐review and editing. **Jiyu Zhang**: Supervision.

## Ethics Statement

All animal experiments were carried out in accordance with the recommendations in the Guide for the Care and Use of Laboratory Animals of the Ministry of Science and Technology of the People's Republic of China, and all efforts were made to minimize suffering.

## Conflicts of Interest

The authors declare no conflicts of interest.

### Peer Review

The peer review history for this article is available at https://publons.com/publon/10.1002/vms3.70361.

## Data Availability

The datasets that support the findings of this study are available from the corresponding author upon reasonable request.
